# The Montreal Cognitive Assessment (MoCA): updated norms and psychometric insights into adaptive testing from healthy individuals in Northern Italy

**DOI:** 10.1007/s40520-021-01943-7

**Published:** 2021-07-27

**Authors:** Edoardo Nicolò Aiello, Chiara Gramegna, Antonella Esposito, Valentina Gazzaniga, Stefano Zago, Teresa Difonzo, Ottavia Maddaluno, Ildebrando Appollonio, Nadia Bolognini

**Affiliations:** 1 School of Medicine and Surgery, University of Milano-Bicocca, Monza, Italy; 2 PhD Program in Neuroscience, University of Milano-Bicocca, Monza, Italy; 3 Department of Psychology, and NeuroMi - Milan Center for Neuroscience, University of Milano Bicocca, Milan, Italy; 4 Fondazione IRCCS Ca’ Granda Ospedale Maggiore Policlinico, University of Milan, Milan, Italy; 5 Department of Psychology, Sapienza University of Rome, Rome, Italy; 6 Milan Center for Neuroscience (NeuroMI), Milan, Italy; 7 Neurology Section, School of Medicine and Surgery, University of Milano-Bicocca, Monza, Italy; 8 Laboratory of Neuropsychology, IRCCS Istituto Auxologico Italiano, Milan, Italy

**Keywords:** Montreal Cognitive Assessment, Cognitive impairment, Item Response Theory, Adaptive testing, Normative data, Cultural differences

## Abstract

**Background:**

The availability of fine-grained, culture-specific psychometric outcomes can favor the interpretation of scores of the Montreal Cognitive Assessment (MoCA), the most frequently used instrument to screen for mild cognitive dysfunctions in both instrumental and non-instrumental domains. This study thus aimed at providing: (i) updated, region-specific norms for the Italian MoCA, by also (ii) comparing them to pre-existing ones with higher geographical coverage; (iii) information on sensitivity and discriminative capability at the item level.

**Methods:**

Five hundred and seventy nine healthy individuals from Northern Italy (208 males, 371 females; age: 63.4 ± 15, 21–96; education: 11.3 ± 4.6, 1–25) were administered the MoCA. Item Response Theory (IRT) was adopted to assess item difficulty and discrimination. Normative values were derived by means of the Equivalent Scores (ESs) method, applied to the MoCA and its sub-scales. Average ESs were also computed. Agreement with previous ESs classification was assessed via Cohen’s *k*.

**Results:**

Age and education significantly predicted all MoCA measures except for Orientation, which was related to age only. No sex differences were detected when tested along with age and education. Substantial disagreements with previous ESs classifications were detected. Several items proved to be scarcely sensitive, especially the *place* item from Orientation and the letter detection task. Memory items showed high discriminative capability, along with certain items assessing executive functions and orientation.

**Discussion:**

Item-level information herewith provided for the Italian MoCA can help interpret its scores by Italian practitioners. Italian practitioners should consider an adaptive use of region-specific norms for the MoCA.

## Introduction

Cognitive screening/first-level tests allow an estimate of global efficiency/functioning by adequately balancing between informativity and practicality of usage [1]. Compared to screening tests for dementia [2], those aimed at detecting mild-to-moderate cognitive impairment [3] may be harder for practitioners to interpret because of (a) the magnitude of the target construct (i.e., the deficit) being less obvious and (b) the amount of information provided by the test being limited [4]. Fine-grained, adaptive psychometric approaches can thus help solve interpretation issues to facilitate diagnostic processes by magnifying informativity [5, 6].

The Montreal Cognitive Assessment (MoCA) [7] is one of the most widespread and psychometrically robust screening tools for cognitive impairments of graded severity [8]. The MoCA is a rapid (5–10’) screening test which evaluates both non-instrumental (executive functioning, attention) and instrumental (language, memory, visuo-spatial abilities, orientation) domains.

In Italy, the MoCA has been adapted and standardized—and both its statistical properties and clinical usability thoroughly examined [9–12].

Psychometric investigations on the MoCA have been carried out both at the sub-test and the single-item levels [13, 14]. A widespread approach that allows a flexible use of cognitive screening tests [15] is to provide norms for their domain-specific sub-tests [10]. Moreover, information regarding single items can further help practitioners interpret test scores by qualitatively assigning different weights to different items [16]. To this last end, Item Response Theory (IRT) analyses [17] have been conducted on MoCA items to assess both their sensitivity and discriminative capability [18–21]. IRT-based analyses indeed proved to yield relevant insights to performance interpretations; for instance, executive- and memory-related items were often shown to be highly informative [18, 19].

Further improvements to adaptive testing may come from deriving norms that account for inter-regional socio-demographic heterogeneity [22]. Cultural differences within a same country have been indeed highlighted as a relevant confounding predictor when interpreting test scores [23].

Therefore, providing region-/culture-specific psychometric fine-grained outcomes and normative data can ameliorate I-level cognitive testing in both clinical and research contexts [24].

It is furthermore worth highlighting that rapid socio-demographic changes may pose additional challenges to practitioners when drawing up-to-date clinical inferences since norms need to be frequently renewed [25].

The present study thus aimed at: (i) providing updated, region-specific normative data for the Italian MoCA and its sub-tests; (ii) comparing existing norms for the MoCA in the Italian population to those drawn from a region-specific Italian sample; (iii) providing IRT-based information regarding sensitivity and discriminative capability of MoCA items in an Italian population sample.

## Methods

### Participants

Five hundred and seventy nine healthy Italian native speakers were recruited in Lombardy, Northern Italy. Exclusion criteria were: (a) a confirmed diagnosis of neurological or psychiatric disorders; (b) general medical conditions possibly affecting cognition (i.e., non-compensated and/or severe metabolic/internal morbidities and systemic/organ failures); (c) intake of psychotropic drugs. Participants suffering from well-compensated metabolic/internal conditions were included [9, 10]. Participants had normal or corrected-to-normal vision and/or hearing. Sample stratification is reported in Table 1. Data were derived from three different normative studies where the MoCA was administered cognitive screening aims; the MoCA was administered as the first test in every study, adopting the same procedure (as detailed below), the same sampling criteria (as detailed above) and geographical coverage. All of these studies were approved by the Research Evaluation Committee of the Department of Psychology of University of Milano-Bicocca on behalf of the Ethical Committee of the same Institution. Participants provided informed consent and signed a data treatment disclaimer for research purposes.

**Table 1:** Sample stratification for age, education and sex

M/F	Age							
Education	35 ≤	36–45	46–55	56–65	66–75	76–85	86–95	≥ 96
4 ≤	0/0	0/0	0/0	0/0	0/0	2/4	0/1	1/0
5–8	0/1	5/2	7/18	13/16	14/41	25/64	5/14	1/0
9–13	6/4	5/10	16/41	33/38	13/7	9/17	2/6	0/0
14–17	1/6	0/4	8/13	15/16	3/2	1/2	1/3	0/0
18–20	3/2	1/4	4/8	11/15	1/2	1/1	0/1	0/0
≥ 21	0/0	0/3	0/3	2/1	0/0	0/0	0/1	0/0

Cells show male/female ratio for each co-occurrence

### Materials

The Italian version of the MoCA was administered to all participants [26]. Items were grouped as follows: Executive Functioning (EF): Trail-Making B (TMT), phonemic fluency and verbal abstraction tasks; Attention (A): serial backward subtraction, letter detection by tapping and forward/backward digit span tasks; Language (L): confrontation naming and sentence repetition ; Visuo-spatial (VS): three-dimension cube copy and Clock Drawing task (CDT); Orientation (O) and Memory (M): spatio-temporal orientation and delayed recall (DR) items, respectively [9, 10].

### Statistical analyses

Normality checks on raw variables were performed descriptively, by evaluating skewness and kurtosis values, and graphically, by visually inspecting histograms and quantile-quantile plots) [27, 28]. Between-variables associations were thus tested via either parametric (Pearson’s) or non-parametric (Spearman’s) techniques. Sex differences were tested *via* independent sample *t *tests.

MoCA reliability was assessed *via* an internal consistency analysis (Cronbach’s α), whereas construct validity by means of a Principal Component Analysis (PCA). Single-item-level analyses were performed by applying a two-parameter logistic IRT model for dichotomous outcomes *via* the R package *ltm* [29]; item difficulty and discrimination were thus computed [17, 30, 31]. Higher values of both parameters correspond to higher levels of the target construct. Cognitive efficiency was regarded as the latent trait.

Regression-based norms were derived *via* the Equivalent Scores (ESs) method [32, 33]; outer and inner tolerance limits (oTL and iTL, respectively) as well as ESs threshold were computed. Average ESs (AESs) [34] were also calculated by averaging ESs of each sub-test to provide a standardized across-domain global index.

Agreement between the present ES classification and those from previous normative studies [9, 10] was tested by crossing level of abilities *via* Cohen’s *k*.

Analyses regarding MoCA total scores were performed on the whole sample, whereas those for single sub-tests and items were conducted on *N* = 535 participants only due to imputation issues.

Statistical power was computed *a posteriori* based on the final multiple regression model (*df*_numerator_ = 3) [35] on MoCA total scores *via* the R package *pwr* [36]—according to previous normative studies [37, 38] and by taking into account *α* = 0.05 and *f*^2^ derived from fit measures.

Analyses were performed *via* SPSS 27 [39] and R 3.6.3 [40]. ES-related procedures were carried out according to guidelines reported by Aiello and Depaoli [41].

## Results

Participants’ demographics and MoCA scores (*M* ± *SD* and *range*) are reported in Table 2.

**Table 2:** Participants’ demographics and cognitive variables

Sex (M/F)	Age (years)	Education (years)	MoCA (*N* = 579)	MoCA-VS (*N* = 535)	MoCA-EF (*N* = 535)	MoCA-L (*N* = 535)	MoCA-A (*N* = 535)	MoCA-M (*N* = 535)	MoCA-O (*N* = 535)
208/371	63.44 ± 15.04 (21–96)	11.27 ± 4.6 (1–25)	24.17 ± 3.93 (8–30)	3.1 ± .97 (0–4)	2.94 ± 1.12 (0–4)	4.48 ± .73 (1–5)	5.4 ± .91 (1–6)	2.33 ± 1.81 (0–5)	5.9 ± 0.5 (2–6)

MoCA, Montreal Cognitive Assessment; VS, visuo-spatial; EF, executive functioning; L, language; A, attention; M, memory; O, orientation. Continuous outcomes are reported as *M* ± *SD* and *range* (in brackets)

Age proved to be inversely related to both total (Spearman’s *r*_s_(579) = − 0.57; *p* < 0.001) and sub-test (−0.46 ≤ *r*_s_(535) ≤ −0.11; .014 ≤ *p* < 0.001) MoCA scores, whereas a positive association with education was found for all measures: MoCA total (*r*_s_(579) = 0.55; *p* < 0.001) and sub-test (0.15 ≤ *r*_s_(535) ≤ 0.53; *p*≤.001) scores. Sex differences were detected with respect to MoCA-A (*t*(441.8) = 2.42; *p* = 0.021; males: 5.52±.81; females: 5.33 ± 0.95), -L (*t*(482.98) = 2.96; *p* = 0.003; males: 4.6 ± 0.6; females: 4.42 ± 0.79) and -VS (*t*(533) = 2.12; *p*=.034; males: 3.22 ± 0.92; females: 3.03 ± 0.98) scores. Moreover, males (24.57±3.47) scored slightly higher (*t*(494.4) = 1.96; *p* = 0.05) than females (23.94 ± 4.15) on the MoCA-total. However, when simultaneously tested, only age and education proved to be significantly predictive of all MoCA measures (age: |0.19| ≤ *β* ≤ |0.38|; *p* < 0.001; education: |0.16| ≤ *β* ≤ |0.42|; *p* < 0.001); however, MoCA-O was found to be predicted by age only (*β* = 0.19; *p* < 0.001). Achieved power was estimated at 1−β ≈ 1, with an effect size *f*^2^ = *R*^2^/(1−*R*^2^) = 0.45/(1−0.45)  = 0.82.

Adjustment equations and grids as well as TLs and ESs thresholds are reported in Tables 3 and 4, respectively. Since both MoCA-M TLs corresponded to negative values, the observation corresponding to the first positive adjusted score was regarded as an empirical iTL (yielding a *p* > 0.99 that 95% of the population performs above it). No adjusted score was thus classified as ES = 0.

**Table 3:** Adjustment grids according to age and education for MoCA total and sub-test raw scores

Sub-test	Education	Age												
		35	40	45	50	55	60	65	70	75	80	85	90	95
Total	5	0.35	0.52	0.73	1	1.34	1.73	2.2	2.75	3.38	4.1	4.92	5.84	6.86
	8	− 1.22	− 1.05	− 0.83	− 0.56	− 0.23	.17	.64	1.18	1.81	2.53	3.35	4.27	5.3
	11	−2.28	− 2.11	− 1.89	− 1.62	− 1.29	− 0.89	− 0.43	0.12	0.75	1.47	2.29	3.21	4.24
	13	− 2.84	− 2.67	− 2.45	− 2.18	− 1.85	− 1.45	− 0.98	− 0.43	0.2	0.92	1.73	2.65	3.68
	16	− 3.53	− 3.36	− 3.14	− 2.87	− 2.54	− 2.14	− 1.67	− 1.13	− 0.5	0.23	1.04	1.96	2.99
	18	− 3.92	− 3.75	− 3.53	− 3.26	− 2.93	− 2.53	− 2.07	− 1.52	− 0.89	− 0.17	0.65	1.57	2.6
	21	− 4.43	− 4.26	− 4.05	− 3.78	− 3.45	− 3.05	− 2.58	− 2.03	− 1.4	− 0.68	0.14	1.06	2.08
VS	5	–	0.06	0.13	0.2	0.28	0.37	0.47	0.57	0.69	0.81	0.93	1.07	1.21
	8	− 0.3	− 0.24	− 0.18	− 0.1	− 0.02	0.07	0.17	0.27	0.38	0.5	0.63	0.77	0.91
	11	− 0.51	− 0.45	− 0.38	− 0.31	− 0.23	− 0.14	− 0.04	0.06	0.18	0.3	0.42	0.56	0.7
	13	− 0.61	− 0.55	− 0.49	− 0.42	− 0.33	− 0.24	− 0.15	− 0.04	0.07	0.19	0.32	0.45	0.6
	16	− 0.75	− 0.69	− 0.62	− 0.55	− 0.47	− 0.38	− 0.28	− 0.18	− 0.07	0.05	0.18	0.32	0.46
	18	− 0.82	− 0.77	− 0.7	− 0.63	− 0.54	− 0.46	− 0.36	− 0.25	− 0.14	− 0.02	0.11	0.24	0.39
	21	− 0.92	− 0.86	− 0.8	− 0.73	− 0.64	− 0.55	− 0.46	− 0.35	− 0.24	− 0.12	0.01	0.14	0.29
EF	5	0.22	0.26	0.31	0.38	0.46	0.56	0.68	0.82	0.97	1.15	1.36	1.59	1.84
	8	− 0.25	− 0.21	− 0.16	− 0.09	− 0.01	0.09	0.21	0.35	0.51	0.69	0.89	1.12	1.38
	11	− 0.57	− 0.53	− 0.47	− 0.41	− 0.32	− 0.22	− 0.11	0.03	0.19	0.37	0.57	0.8	1.06
	13	− 0.74	− 0.69	− 0.64	− 0.57	− 0.49	− 0.39	− 0.27	− 0.14	0.02	0.2	0.41	0.64	0.89
	16	− 0.94	− 0.9	− 0.85	− 0.78	− 0.7	− 0.6	− 0.48	− 0.34	− 0.19	− 0.01	0.2	0.43	0.69
	18	− 1.06	− 1.02	− 0.96	− 0.9	− 0.81	− 0.71	− 0.6	− 0.46	− 0.3	− 0.12	0.08	0.31	0.57
	21	− 1.21	− 1.17	− 1.12	− 1.05	− 0.97	− 0.87	− 0.75	− 0.61	− 0.46	− 0.28	− 0.07	0.16	0.41
L	5	0.12	0.14	0.16	0.19	0.22	0.26	0.31	0.37	0.44	0.51	0.6	0.69	0.8
	8	− 0.15	− 0.14	− 0.11	− 0.09	− 0.05	− 0.01	0.04	0.1	0.16	0.24	0.32	0.42	0.53
	11	− 0.28	− 0.26	− 0.24	− 0.21	− 0.18	− 0.13	− 0.08	− 0.03	0.04	0.11	0.2	0.3	0.40
	13	− 0.33	− 0.31	− 0.29	− 0.26	− 0.23	− 0.18	− 0.14	− 0.08	− 0.01	0.06	0.15	0.24	0.35
	16	− 0.38	− 0.36	− 0.34	− 0.31	− 0.28	− 0.24	− 0.19	− 0.13	− 0.06	0.01	0.1	0.19	0.3
	18	− 0.41	− 0.39	− 0.37	− 0.34	− 0.3	− 0.26	− 0.21	− 0.16	− 0.09	− 0.01	0.07	0.17	0.27
	21	− 0.44	− 0.42	− 0.4	− 0.37	− 0.33	− 0.29	− 0.24	− 0.19	− 0.12	− .04	0.04	0.14	0.25
A	5	0.09	0.11	0.14	0.17	0.21	0.25	0.31	0.37	0.44	0.52	0.61	0.72	0.84
	8	− 0.12	− 0.1	− 0.07	− 0.04	–	0.04	0.09	0.16	0.23	0.31	0.4	0.51	0.63
	11	− 0.26	− 0.24	− 0.22	− 0.18	− 0.15	− 0.1	− 0.05	0.01	0.09	0.17	0.26	0.37	0.48
	13	− 0.33	− .31	− 0.29	− 0.26	− 0.22	− 0.18	− 0.12	− 0.06	0.01	0.09	0.19	0.29	0.41
	16	− 0.43	− 0.41	− 0.38	− 0.35	− 0.31	− 0.27	− .22	− 0.15	− 0.08	–	0.09	0.2	0.31
	18	− 0.48	− 0.46	− 0.44	− 0.41	− 0.37	− 0.32	− 0.27	− 0.21	− 0.13	− 0.05	0.04	0.14	0.26
	21	− 0.55	− 0.53	− 0.51	− 0.47	− 0.44	− 0.39	− 0.34	− 0.28	− 0.2	− 0.12	0.03	0.08	0.19
M	5	− .56	− 0.43	− 0.29	− 0.13	0.05	0.24	0.45	0.68	0.92	1.18	1.45	1.75	2.06
	8	− 0.8	− 0.68	− 0.53	− 0.37	− 0.2	− 0.01	0.2	0.43	0.67	0.93	1.21	1.5	1.81
	11	− 1	− 0.88	− 0.73	− 0.58	− 0.4	− 0.21	–	0.23	0.47	0.73	1.01	1.3	1.61
	13	− 1.12	− 1	− 0.85	− 0.69	− 0.52	− 0.33	− 0.12	0.11	0.35	0.61	0.89	1.18	1.49
	16	− 1.29	− 1.16	− 1.02	− 0.86	− 0.68	− 0.49	− 0.28	− 0.05	0.19	0.45	0.72	1.02	1.33
	18	− 1.39	− 1.26	− 1.12	− 0.96	− 0.78	− 0.59	− 0.38	− 0.15	0.09	0.35	0.62	0.92	1.23
	21	− 1.53	− 1.4	− 1.26	− 1.1	− 0.92	− 0.73	− 0.52	− 0.29	− 0.05	0.21	0.48	0.78	1.09
O		− 0.13	− 0.11	− 0.09	− 0.08	− 0.06	− 0.03	− 0.01	0.02	0.06	0.1	0.15	0.23	0.37

MoCA, Montreal Cognitive Assessment; VS, visuo-spatial; EF, executive functioning; L, language; A, attention; M, memory; O, orientation; Total: adjusted score** = **raw score + .000008*[(age^3^)-297697.184801] -3.331407*[ln(education)-2.325648]; VS: adjusted score** = **raw score + .000155*[(age^2^)-4168.682243]-0.645622*[ln(education)-2.322051]; EF: adjusted score** = **raw score + .000002*[(age^3^)-290195.728972]-.996668*[ln(education)-2.322051]; L: adjusted score** = **raw score + .00000083757*[(age^3^)- 290195.728972] + 3.645727*[(1/education)-0.110560]; A: adjusted score** = **raw score + .00000091089*[(age^3^)- 290195.728972]-0.448568*[ln(education)-2.322051]; M: adjusted score** = **raw score + .000335*[(age^2^)- 4168.682243]-0.413262*[sqrt(education)-3.276794]; O: adjusted score** = **raw score-0.191626*[ln(100-age)-3.515369]. Significant decimals of adjustment factors are displayed. Adjustment factors have been extracted from the aforementioned formula and do not always reflect empirical co-occurrences

**Table 4:** Equivalent Scores for MoCA total and sub-test adjusted scores

	oTL	iTL	Equivalent Scores				
			0	1	2	3	4
MoCA	18.58	19.48	≤ 18.58	18.59–20.69	20.7–22.56	22.57–24.52	≥ 24.53
MoCA-VS	1.36	1.74	≤ 1.36	1.37–2.03	2.04–2.64	2.65–3.22	≥ 3.23
MoCA-EF	1.07	1.46	≤ 1.07	1.08–1.87	1.88–2.45	2.46–3.07	≥ 3.08
MoCA-L	2.98	3.44	≤ 2.98	2.99–3.71	3.72–4.15	4.16–4.71	≥ 4.72
MoCA-A	3.44	3.79	≤ 3.44	3.45–4.5	4.51–5.09	5.1–5.66	≥ 5.67
MoCA-M*	–	0.11	–	≤ 0.45	.46–1.28	1.29–2.29	≥ 2.3
MoCA-O	4.92	4.97	≤ 4.92	4.93–5.84	5.85–5.93	5.94–5.96	≥ 5.97
MoCA-AES	1.83	2.33	≤ 1.83	–	–	–	–

MoCA, Montreal Cognitive Assessment; VS, visuo-spatial; EF, executive functioning; L, language; A, attention; M, memory; O, orientation; oTL, outer tolerance limit; iTL, inner tolerance limit; AES, Average Equivalent Score. *It is not possible to classify an adjusted score on the MoCA-M as ES = 0. AESs are calculated by averaging ESs of each sub-test to provide a standardized across-domain global index

AESs proved to be independent from sex (*t*(533) = 1.8; *p*=0.073), age (*r*(535)=0.07; *p*=0.119) and education (*r*(535) = 0.03; *p* = 0.44).

Weak agreement (0.17 ≤ *k* ≤ 0.57) [42] was detected between the present and both

Conti et al.’s [9] and Santangelo et al.’s [10] ES classifications (see Table 5). More specifically, ESs allotments here reported proved to be stricter than those of Santangelo et al.’s [10] with regard to MoCA-total, -VS, -EF and -A, whereas less strict with regard to and -O and Conti et al.’s [9] total.

**Table 5:**
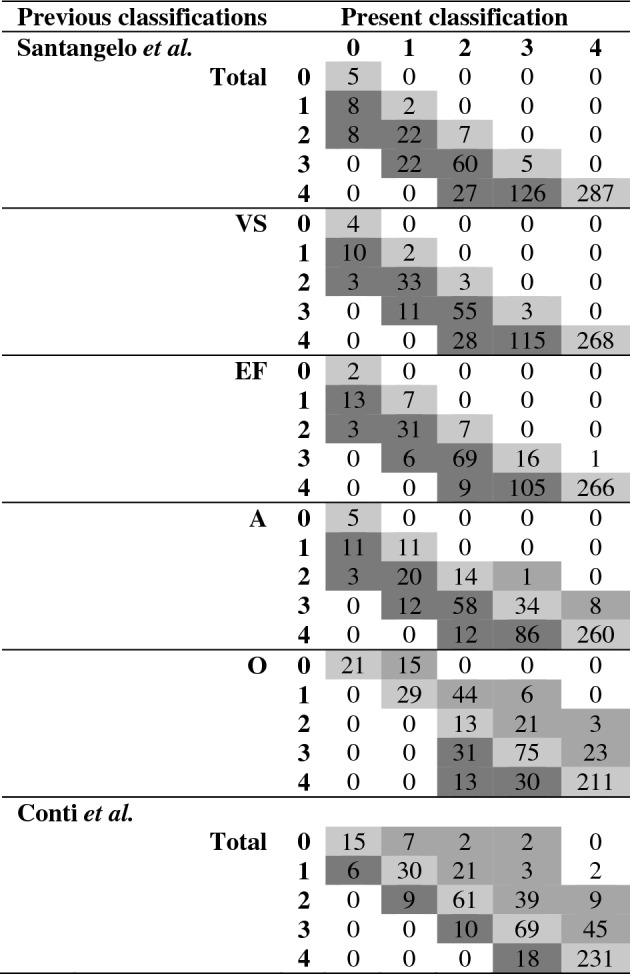
Comparison between Equivalent Scores classifications

MoCA, Montreal Cognitive Assessment; VS, visuo-spatial; EF, executive functioning; A, attention; M, memory; O, orientation. Diagonal co-occurrences index inter-rater agreements; extra-diagonal co-occurrences index disagreements (below the diagonal: the present classification is more conservative; above the diagonal: the present classification is less conservative). Language sub-test could not be compared due to different ranges

As regards item-level analyses, the MoCA proved to be internally consistent (Cronbach’s *α* = 0.81).

A mono-component factor (15.9% of variance explained) structure was selected from PCA, with the majority of items highly loading (0.3 ≤ *r* ≤ 0.55), except for *N* = 8 items (CDT contour, digit span backward, *lion* and *camel* naming and all MoCA-O items except for *year*; .02 ≤ *r* ≤ 0.26).

Item difficulty and discrimination values are displayed in Table 6. The most difficult items proved to be the three-dimension cube copy, CDT hands, repetition of the second sentence, phonemic fluency, the second verbal abstraction item and DR items. The least difficult ones were CDT contour, *lion*-naming, the letter detection task and *month*, *place* and *city* items of MoCA-O. TMT, repetition of the first sentence, DR items and *year* and *city* of MoCA-O proved to be the most effective in discriminating between different levels of ability, whilst those with the lowest values of discrimination were *place* of MoCA-O and the letter detection task.

**Table 6:** Item difficulty and discrimination for the MoCA

Item	Difficulty	Discrimination
TMT	− 1.347	1.527^c^
Cube	− 0.892	1.247
CDT-C	− 5.416^b^	0.697
CDT-N	− 1.505	0.64
CDT-H	− 0.946	1.331
Lion	− 12^b^	0.44
Rhino	− 1.86	1.373
Camel	− 3.803^a^	1.333
FDS	− 2.611^a^	0.827
BDS	− 3.19^a^	0.644
A	− 11.22^b^	0.221
93	− 3.299^a^	1.126
86	− 1.778	0.812
79	− 2.243^a^	0.904
72	− 1.856	1.108
65	− 2.037^a^	0.934
Rep. 1	− 2.676^a^	1.691^c^
Rep. 2	− 0.961	0.862
Flu	− 0.862	1.1
Abst. 1	− 1.55	1.606^c^
Abst. 2	− 0.488	0.724
DR 1	0.56	1.663^c^
DR 2	0.064	1.919^d^
DR 3	− 0.05	1.446^a^
DR 4	0.325	1.962^d^
DR 5	− 0.162	1.863^d^
Date	− 2.993^a^	1.022
Month	− 5.984^b^	0.772
Year	− 3.347^a^	1.512^c^
Day	− 3.68	1.22
Place	− 124^b^	0.034
City	− 4.157^b^	1.732^d^

MoCA, Montreal Cognitive Assessment; TMT, Trail Making Test; CDT, Clock Drawing Test; -C, contour; -N, numbers; -H, hands; FDS, forward digit span; BDS, backward digit span; A, letter detection task; Rep., sentence repetition; Flu., phonemic fluency; Abst., abstraction task; DR, delayed recall. Higher values correspond to higher difficulty and discriminative capability of items. ^a^low difficulty; ^b^very low difficulty**;** ^c^high discrimination; (Hambleton et al*.* [30]) ^d^very high discrimination (Baker and Kim [31]). Very low difficulty items (≤ − 4) were identified by doubling the “cut-off” value for “very easy” items (≤ − 2) established by Hambleton et al*.* [30]

## Discussion

The present work provides Italian practitioners with updated, region-specific normative data for the MoCA, as well as with IRT-based, item-level information that may allow a more flexible and informative use of this screening instrument.

Although norms for the Italian MoCA have been provided in previous studies [9, 10], recent changes in demographic composition and socio-cultural features of Italian population motivated the normative branch of this study. Moreover, the present sample covers wider ranges of age and education and is larger (*N* = 579; age: 21–96; education: 1–25) when compared to previous normative studies - Conti et al. [9]: *N* = 225; age: 60–80; education: 5–23; Santangelo et al. [10]: *N* = 415; age: 21–95; education: 1–21. Norms here reported are thus likely to be more representative and generalizable as far as sample size and coverage of anagraphic–demographic variables are concerned.

Moreover, the oTL for MoCA-M had not been provided by Santangelo et al. [10] because it corresponded to a negative adjusted score. Nonetheless, despite this finding having been replicated also in the present study, an empirical iTL for MoCA-M has been with provided, along with ESs thresholds (which, however, did not correspond to negative adjusted scores). Although caution is needed when interpreting this iTL, its practical use is quite intuitive. For instance, only for young and highly educated individuals a raw score of 1 would be classified as below the aforementioned iTL. Thereupon, practitioners would not be allowed to judge that a score below the MoCA-M iTL falls in the worst 5% of the population, although it would be possible to say that 99% of healthy individuals perform above it.

With respect to anagraphic–demographic predictors, MoCA-O scores proved not to be influenced by education in the present study. This finding diverges from previous ones regarding not only the MoCA [10], but also other cognitive screening tests [43, 44]. Similarly, although males were found as performing better than females on MoCA-A and -VS, when sex was tested individually, no such differences have been yielded from models additionally accounting for age and education, contrarily to Santangelo et al.’s [10] study. This finding was also true for MoCA-L, although it has not been previously reported [10]. This discrepancy may be attributed to age/education voiding sex differences in this larger sample, and it is in line with inconsistent findings in concerning literature [45].

Along with the above inconsistencies regarding anagraphic–demographic variables, the fact that the present cut-off thresholds happened to systematically diverge from those of Conti et al*.* [9] and Santangelo et al*.* [10] is suggestive of relevant inter-regional differences that should be taken into consideration by Northern Italian practitioners [46]. It is noteworthy that this last aspect has been recently addressed in Italy with respect to the Mini-Mental State Examination [47], for whom region-specific norms have been recently provided for Southern Italian individuals.

Major contributions to an adaptive interpretation [48, 49] of the Italian MoCA also come from single-item-level analyses, which indicate the need to pay particular attention to highly discriminative items when specificity has to be favored, and to highly difficult ones when sensitivity does. Of relevance, despite cultural/language differences [24], the present findings are in line with previous ones from eastern countries with regard to the high discriminative capability of MoCA-EF and -M items [18, 19].

This work has a main limitation that needs consideration: a different cognitive screening test was not administered since it was out of the present aims to assess concurrent/convergent validity of the MoCA. However, due to the lack of such data, it is not possible to rule out sub-clinical cognitive deficits in participants. It is also noteworthy that item- and sub-test-level analyses were performed on a smaller sample (535 out of 579 participants) due to completely-at-random missing values [50].

In conclusion, the present study and its results favor a more informative and flexible use, scoring and interpretation of the Italian MoCA by providing updated and region-specific normative data at the sub-test level, also comprising a proxy cut-off for MoCA-M scores; moreover, novel information on sensitivity and discriminative capability of single Italian MoCA items have been provided.

## Data Availability

Data collected and analyzed during the present study are available on the Open Science Framework (OSF) repository (https://osf.io/cykbe/).
