# Correction to: The Montreal Cognitive Assessment (MoCA): updated norms and psychometric insights into adaptive testing from healthy individuals in Northern Italy

**DOI:** 10.1007/s40520-022-02161-5

**Published:** 2022-05-25

**Authors:** Edoardo Nicolò Aiello, Chiara Gramegna, Antonella Esposito, Valentina Gazzaniga, Stefano Zago, Teresa Difonzo, Ottavia Maddaluno, Ildebrando Appollonio, Nadia Bolognini

**Affiliations:** 1 School of Medicine and Surgery, University of Milano-Bicocca, Monza, Italy; 2 PhD Program in Neuroscience, University of Milano-Bicocca, Monza, Italy; 3 Department of Psychology, University of Milano-Bicocca, Milan, Italy; 4 Fondazione IRCCS Ca’ Granda Ospedale Maggiore Policlinico, University of Milan, Milan, Italy; 5 Department of Psychology, Sapienza University of Rome, Rome, Italy; 6 Milan Center for Neuroscience (NeuroMI), Milan, Italy; 7 Neurology Section, School of Medicine and Surgery, University of Milano-Bicocca, Monza, Italy; 8 Department of Psychology, and NeuroMi-Milan Center for Neuroscience, University of Milano Bicocca, Milan, Italy; 9 Laboratory of Neuropsychology, IRCCS Istituto Auxologico Italiano, Milan, Italy

## Correction to: Aging Clinical and Experimental Research (2021) 34:375–382 10.1007/s40520-021-01943-7

In the original version of this article, the affiliation details for Author Nadia Bolognini were incorrectly given as below.

- Department of Psychology, University of Milano-Bicocca, Milan, Italy
- Milan Center for Neuroscience (NeuroMI), Milan, Italy
- Neuropsychological Laboratory, IRCCS Istituto Auxologico Italiano, Milan, Italy

but should have been as below.

- Department of Psychology, and NeuroMi-Milan Center for Neuroscience, University of Milano Bicocca, Milan, Italy
- Laboratory of Neuropsychology, IRCCS Istituto Auxologico Italiano, Milan, Italy

The original article has been updated.

