# Correction to: The Montreal Cognitive Assessment (MoCA): updated norms and psychometric insights into adaptive testing from healthy individuals in Northern Italy

**DOI:** 10.1007/s40520-023-02509-5

**Published:** 2023-08-08

**Authors:** Edoardo Nicolò Aiello, Chiara Gramegna, Antonella Esposito, Valentina Gazzaniga, Stefano Zago, Teresa Difonzo, Ottavia Maddaluno, Ildebrando Appollonio, Nadia Bolognini

**Affiliations:** 1 School of Medicine and Surgery, University of Milano-Bicocca, Monza, Italy; 2 PhD Program in Neuroscience, University of Milano-Bicocca, Monza, Italy; 3 Department of Psychology, University of Milano-Bicocca, Milan, Italy; 4 Fondazione IRCCS Ca’ Granda Ospedale Maggiore Policlinico, University of Milan, Milan, Italy; 5 Department of Psychology, Sapienza University of Rome, Rome, Italy; 6 Milan Center for Neuroscience (NeuroMI), Milan, Italy; 7 Neurology Section, School of Medicine and Surgery, University of Milano-Bicocca, Monza, Italy; 8 Neuropsychological Laboratory, IRCCS Istituto Auxologico Italiano, Milan, Italy

**Correction to: Aging Clinical and Experimental Research (2022) 34:375–382** 10.1007/s40520-021-01943-7

In the original version of this article, some typos regarding the adjustment coefficients for raw MoCA-Visuo-spatial (MoCA-VS) and MoCA-Attention (MoCA-A) scores (Table 3) were present and have now been amended (amended MoCA-VS cell: age=35, education=8; amended MoCA-A cells: age=55, education=8; age=35, education=13). Moreover, some imprecisions regarding the discussion of the discrepancies between the present and previous normative studies have been amended (page 379, Discussion: "More specifically, ESs allotments here reported proved to be stricter than those of Santangelo et al.’s [1] with regard to MoCA-total, -VS, -EF and -A, whereas less strict with regard to MoCA-O and Conti et al.’s [2] total"). We are thankful to the Researcher that has drawn our attention on such elements.

The updated Table 3 is below:

**Table 3:** Adjustment grids according to age and education for MoCA total and subtest raw scores

Sub-test	Education	Age												
		35	40	45	50	55	60	65	70	75	80	85	90	95
Total	5	0.35	0.52	0.73	1	1.34	1.73	2.2	2.75	3.38	4.1	4.92	5.84	6.86
	8	− 1.22	− 1.05	− 0.83	− 0.56	− 0.23	0.17	0.64	1.18	1.81	2.53	3.35	4.27	5.3
	11	− 2.28	− 2.11	− 1.89	− 1.62	− 1.29	− 0.89	− 0.43	0.12	0.75	1.47	2.29	3.21	4.24
	13	− 2.84	− 2.67	− 2.45	− 2.18	− 1.85	− 1.45	− 0.98	− 0.43	0.2	0.92	1.73	2.65	3.68
	16	− 3.53	− 3.36	− 3.14	− 2.87	− 2.54	− 2.14	− 1.67	− 1.13	− 0.5	0.23	1.04	1.96	2.99
	18	− 3.92	− 3.75	− 3.53	− 3.26	− 2.93	− 2.53	− 2.07	− 1.52	− 0.89	− 0.17	0.65	1.57	2.6
	21	− 4.43	− 4.26	− 4.05	− 3.78	− 3.45	− 3.05	− 2.58	− 2.03	− 1.4	− 0.68	0.14	1.06	2.08
VS	5	−	0.06	0.13	0.2	0.28	0.37	0.47	0.57	0.69	0.81	0.93	1.07	1.21
	8	− 0.3	− 0.24	− 0.18	− 0.1	− 0.02	0.07	0.17	0.27	0.38	0.5	0.63	0.77	0.91
	11	− 0.51	− 0.45	− 0.38	− 0.31	− 0.23	− 0.14	− 0.04	0.06	0.18	0.3	0.42	0.56	0.7
	13	− 0.61	− 0.55	− 0.49	− 0.42	− 0.33	− 0.24	− 0.15	− 0.04	0.07	0.19	0.32	0.45	0.6
	16	− 0.75	− 0.69	− 0.62	− 0.55	− 0.47	− 0.38	− 0.28	− 0.18	− 0.07	0.05	0.18	0.32	0.46
	18	− 0.82	− 0.77	− 0.7	− 0.63	− 0.54	− 0.46	− 0.36	− 0.25	− 0.14	− 0.02	0.11	0.24	0.39
	21	− 0.92	− 0.86	− 0.8	− 0.73	− 0.64	− 0.55	− 0.46	− 0.35	− 0.24	− 0.12	0.01	0.14	0.29
EF	5	0.22	0.26	0.31	0.38	0.46	0.56	0.68	0.82	0.97	1.15	1.36	1.59	1.84
	8	− 0.25	− 0.21	− 0.16	− 0.09	− 0.01	0.09	0.21	0.35	0.51	0.69	0.89	1.12	1.38
	11	− 0.57	− 0.53	− 0.47	− 0.41	− 0.32	− 0.22	− 0.11	0.03	0.19	0.37	0.57	0.8	1.06
	13	− 0.74	− 0.69	− 0.64	− 0.57	− 0.49	− 0.39	− 0.27	− 0.14	0.02	0.2	0.41	0.64	0.89
	16	− 0.94	− 0.9	− 0.85	− 0.78	− 0.7	− 0.6	− 0.48	− 0.34	− 0.19	− 0.01	0.2	0.43	0.69
	18	− 1.06	− 1.02	− 0.96	− 0.9	− 0.81	− 0.71	− 0.6	− 0.46	− 0.3	− 0.12	0.08	0.31	0.57
	21	− 1.21	− 1.17	− 1.12	− 1.05	− 0.97	− 0.87	− 0.75	− 0.61	− 0.46	− 0.28	− 0.07	0.16	0.41
L	5	0.12	0.14	0.16	0.19	0.22	0.26	0.31	0.37	0.44	0.51	0.6	0.69	0.8
	8	− 0.15	− 0.14	− 0.11	− 0.09	− 0.05	− 0.01	0.04	0.1	0.16	0.24	0.32	0.42	0.53
	11	− 0.28	− 0.26	− 0.24	− 0.21	− 0.18	− 0.13	− 0.08	− 0.03	0.04	0.11	0.2	0.3	0.40
	13	− 0.33	− 0.31	− 0.29	− 0.26	− 0.23	− 0.18	− 0.14	− 0.08	− 0.01	0.06	0.15	0.24	0.35
	16	− 0.38	− 0.36	− 0.34	− 0.31	− 0.28	− 0.24	− 0.19	− 0.13	− 0.06	0.01	0.1	0.19	0.3
	18	− 0.41	− 0.39	− 0.37	− 0.34	− 0.3	− 0.26	− 0.21	− 0.16	− 0.09	− 0.01	0.07	0.17	0.27
	21	− 0.44	− 0.42	− 0.4	− 0.37	− 0.33	− 0.29	− 0.24	− 0.19	− 0.12	− 0.04	0.04	0.14	0.25
A	5	0.09	0.11	0.14	0.17	0.21	0.25	0.31	0.37	0.44	0.52	0.61	0.72	0.84
	8	− 0.12	− 0.1	− 0.07	− 0.04	–	0.04	0.09	0.16	0.23	0.31	0.4	0.51	0.63
	11	− 0.26	− 0.24	− 0.22	− 0.18	− 0.15	− 0.1	− 0.05	0.01	0.09	0.17	0.26	0.37	0.48
	13	− 0.33	− 0.31	− 0.29	− 0.26	− 0.22	− 0.18	− 0.12	− 0.06	0.01	0.09	0.19	0.29	0.41
	16	− 0.43	− 0.41	− 0.38	− 0.35	− 0.31	− 0.27	− 0.22	− 0.15	− 0.08	–	0.09	0.2	0.31
	18	− 0.48	− 0.46	− 0.44	− 0.41	− 0.37	− 0.32	− 0.27	− 0.21	− 0.13	− 0.05	0.04	0.14	0.26
	21	− 0.55	− 0.53	− 0.51	− 0.47	− 0.44	− 0.39	− 0.34	− 0.28	− 0.2	− 0.12	0.03	0.08	0.19
M	5	− 0.56	− 0.43	− 0.29	− 0.13	0.05	0.24	0.45	0.68	0.92	1.18	1.45	1.75	2.06
	8	− 0.8	− 0.68	− 0.53	− 0.37	− 0.2	− 0.01	0.2	0.43	0.67	0.93	1.21	1.5	1.81
	11	− 1	− 0.88	− 0.73	− 0.58	− 0.4	− 0.21	–	0.23	0.47	0.73	1.01	1.3	1.61
	13	− 1.12	− 1	− 0.85	− 0.69	− 0.52	− 0.33	− 0.12	0.11	0.35	0.61	0.89	1.18	1.49
	16	− 1.29	− 1.16	− 1.02	− 0.86	− 0.68	− 0.49	− 0.28	− 0.05	0.19	0.45	0.72	1.02	1.33
	18	− 1.39	− 1.26	− 1.12	− 0.96	− 0.78	− 0.59	− 0.38	− 0.15	0.09	0.35	0.62	0.92	1.23
	21	− 1.53	− 1.4	− 1.26	− 1.1	− 0.92	− 0.73	− 0.52	− 0.29	− 0.05	0.21	0.48	0.78	1.09
O		− 0.13	− 0.11	− 0.09	− 0.08	− 0.06	− 0.03	− 0.01	0.02	0.06	0.1	0.15	0.23	0.37

Total: adjusted score = raw score + 0.000008*[(age^3) − 297,697.184801] − 3.331407*[ln(education) − 2.325648]; VS: adjusted score = raw score + 0.000155*[(age^2) − 4168.682243] − 0.645622*[ln(education) − 2.322051]; EF: adjusted score = raw score + 0.000002*[(age^3) − 290,195.728972] − 0.996668*[ln(education) − 2.322051]; L: adjusted score = raw score + 0.00000083757*[(age^3) − 290,195.728972] + 3.645727*[(1/education) − 0.110560]; A: adjusted score = raw score + 0.00000091089*[(age^3) − 290,195.728972] − 0.448568*[ln(education) − 2.322051]; M: adjusted score = raw score + 0.000335*[(age^2) − 4168.682243] − 0.413262*[sqrt(education) − 3.276794]; O: adjusted score = raw score − 0.191626*[ln(100 − age) − 3.515369]. Significant decimals of adjustment factors are displayed. Adjustment factors have been extracted from the aforementioned formula and do not always reflect empirical co-occurrences

*MoCA* Montreal Cognitive Assessment, *VS* Visuo-spatial, *EF* Executive Functioning, *L* Language, *A* Attention, *M *Memory, *O* Orientation

The original article has been updated.
